# Review on *PRNP* genetics and susceptibility to chronic wasting disease of *Cervidae*

**DOI:** 10.1186/s13567-021-00993-z

**Published:** 2021-10-07

**Authors:** Katayoun Moazami-Goudarzi, Olivier Andréoletti, Jean-Luc Vilotte, Vincent Béringue

**Affiliations:** 1 University Paris-Saclay, INRAE, AgroParisTech, GABI, 78350 Jouy-en-Josas, France; 2 UMR INRAE ENVT 1225 - IHAP, École Nationale Vétérinaire de Toulouse, 31076 Toulouse, France; 3 University Paris-Saclay, INRAE, UVSQ, VIM, 78350 Jouy-en-Josas, France

**Keywords:** CWD, *Cervidae*, prions, polymorphisms, strains, zoonosis, transgenic mice, host pathogen interaction

## Abstract

To date, chronic wasting disease (CWD) is the most infectious form of prion disease affecting several captive, free ranging and wild cervid species. Responsible for marked population declines in North America, its geographical spread is now becoming a major concern in Europe. Polymorphisms in the prion protein gene (*PRNP*) are an important factor influencing the susceptibility to prions and their rate of propagation. All reported cervid *PRNP* genotypes are affected by CWD. However, in each species, some polymorphisms are associated with lower attack rates and slower progression of the disease. This has potential consequences in terms of genetic selection, CWD diffusion and strain evolution. CWD also presents a zoonotic risk due to prions capacity to cross species barriers. This review summarizes our current understanding of CWD control, focusing on *PRNP* genetic, strain diversity and capacity to infect other animal species, including humans.

## Introduction

Mammalian prions are responsible for six transmissible spongiform encephalopathies (TSEs) in Human; sporadic and familial Creutzfeldt Jakob disease (CJD), variant CJD, Kuru disease, Gerstmann-Straüssler-Scheinker syndrome, fatal familial insomnia and variably protease-sensitive prionopathy. These progressive neurological degenerations are invariably fatal. A key feature in TSE pathogenesis is the accumulation of the host-encoded cellular prion protein (PrP^C^) into a misfolded aggregated conformer PrP^Sc^ that is the principal, if not the sole, constituent of the infectious agent (called prion). TSEs also exist in a wide range of animals, including bovine spongiform encephalopathy (BSE) in cattle, scrapie in sheep and goats, transmissible mink encephalopathy, feline spongiform encephalopathy, camel prion disease, exotic ungulate spongiform encephalopathy and chronic wasting disease (CWD). CWD affects captive, free ranging (semi-domesticated) and wild *Cervidae*.

Following an incubation period of 2–4 years in wild *Cervidae*, CWD-affected animals develop behaviour, sensory and locomotor signs that are pathognomonic of TSEs [1]. Clinical signs include isolation from the herd, listlessness, lowering of head and ears, hyper-excitability, progressive weight loss but also polydipsia, polyuria, ruminal atony, drooling, teeth grinding, and loss of fear of human. At the late stage of the disease, clinical signs include respiratory distress, emaciation, ataxia, depression and weakness. To date, CWD is probably the most infectious TSEs [2]. CWD transmission occurs mostly horizontally by animal contact and the environment due to prion excretion from infected animals. CWD is characterized by an extensive deposition of PrP^Sc^ (termed here PrP^CWD^) and of infectivity in the CNS and in the lymphoid tissue. In addition PrP^CWD^ and infectivity can be discarded in bodily fluids (urine, faeces, saliva), placenta, decomposing carcasses from dead animals and fomites from the suites of infectious deer prions [3–5]. Edible tissues in which PrP^CWD^ has been detected are heart, liver, kidney, tongue, pancreas, blood, adipose tissue, lymphoreticular system and antler velvet [6–11]. An increasing phenomenon of antler cannibalism was recently quantified among the affected reindeer population from Norway and found to potentially contribute to CWD emergence [12]. CWD prions bound to soil components where TSE-infected animals stood, persist for many years (at least 16 years for scrapie sheep [13]) and remain infectious by the oral route of exposure [14]. Variations in soil types and mineralogy, clay and humus content are the main factors responsible for PrP^Sc^ persistence and recovery after prolonged incubation [15, 16]. While, an organic soil component, humic acids, can decrease CWD infectivity [17], soils from meadow regions (montmorillonite, mineralogy and high humus content) show high ability to bind PrP^CWD^ and increased infectivity. A time-dependent decline in recovery of PrP^CWD^ has been found but does not correlate with prion infectivity levels [18]. In addition, after serial protein misfolding cyclic amplification, PrP^CWD^ is detected in environmental water and mineral licks [19, 20]. Thus, human activities, like supplemental feedings, can increase the rate of aggregation and the likelihood of disease transmission. A recent study estimated that the contact rates in Elk population from Wyoming were 2.6 times larger when feeding occurred [21].

## CWD epidemic in North America

CWD has spread into populations of wild *Cervidae.* Its geographic range and prevalence are constantly increasing in North America (up to 4% per year). The USA has, so far, the most widespread CWD infection worldwide, due to its presence for at least 50 years. CWD frequently occurs in domestic animals followed by cases in the wild population thanks to breeding conditions and husbandry systems that allow direct or indirect contact between farm animals and wildlife populations. Furthermore, CWD transmission is more effective in high-density herds and the disease prevalence may be more a function of social and foraging behaviour differences between species. This prevalence typically declines with distance from heavily affected areas and the landscape connectivity plays a major role in the spread of the disease [22]. CWD was first observed in a mule deer (*Odocoileus hemionus*) in a Colorado research facility in 1967. It was discovered in 1981 in wild deer [23, 24]. In Canada, CWD was reported in 1977 at the Toronto Zoo, after importation of CWD-infected animals from a US zoo. A 2006 study by Dubé et al. retrospectively investigated the occurrence of CWD in 105 animals that died at the Toronto Zoo from 1973 to 2003 [25]. CWD was detected in 7 mule deer (died between 77 and 79) and 1 black-tailed deer (died in 1981). In 2000, CWD was detected in a wild mule deer in Saskatchewan, Canada [26]. To date, the disease is present in 26 states of the USA and three Canadian provinces (United States Geological survey, National Wildlife Health Center, updated May 2021). CWD prevalence could reach 79% in captive herds, e.g. White-Tailed Deer (WTD) from south-central Wisconsin [27] and 33% in wild populations, e.g. high-prevalence CWD endemic area like Wyoming. In this hunting area, an intense monitoring study, conducted from 2003 to 2010 via radio-telemetry and global positioning system collars, determined that CWD was the cause of a 10.4% annual decline in free ranging WTD population [28]. In south-eastern Wyoming, average annual CWD prevalence in mule deer exceeds 20% and contributes to a 21% annual population decline [29].

In 2001, epidemiological investigations confirmed that CWD was introduced to the Korean peninsula by captive elk (*Cervus elaphus nelsoni*), imported from Canada in 1994 and 1997 [30, 31]. CWD was subsequently detected in farmed elk populations in 2001, 2004, 2005, 2010 and 2016 and since no evidence of natural CWD transmission to sika deer has been documented [32]. In other countries, CWD was not reported until 2016.

## CWD emergence in Scandinavia

In 2016, following routine surveillance, four CWD cases were documented in wild Eurasian tundra reindeer (*Rangifer tarandus tarandus*) located in the zone 1 of Nordfjella mountain, in southern Norway [33, 34]. Following these cases, an unprecedented CWD eradication campaign was performed between 2016 and 2018. Hunting (*N* = 582), professional marksmen interventions (*N* = 1399) or normal animal deaths (*N* = 43) resulted in the eradication of this entire subpopulation [35]. Analysis of the dead animals for the presence of CWD prions resulted in a 1.6% prevalence in this adult population [36].

The Norwegian wild Eurasian tundra reindeer population is fragmented in 23 separated sub-populations. The above-mentioned eradicated subpopulation constituted approximately 10% of the wild European tundra reindeer population. On September 2020, one more reindeer positive case was identified in a separated population located in Hardangervidda. This region is considered to account for the largest wild Norwegian Eurasian tundra reindeer subpopulation with about 10–11 000 reindeer. To date 14 males and 6 females, aged between 1.5 to 8 years, have been tested positive for CWD in reindeer from Norway. In 2017, one Norwegian red deer (*Cervus elaphus elaphus*) was identified from the 4082 tested. This 16-year-old female was shot by a hunter in October 2017 in the Gjemnes municipality in western Norway and had no signs of disease.

The third CWD positive species identified so far in Scandinavia is the moose (*Alces alces*). Seven cases, aged between 10 to 20 years, were identified in Eastern Norway. These cases were located in Selbu (*N* = 3), Lierne (*N* = 1), Sigdal (*N* = 1), Flesberg (*N* = 1) and Steinkjer (*N* = 1) municipalities [37, 38]. Based on seasonal migrations, it is considered that they likely represent different moose subpopulations [39, 40]. In Easter Finland, one found dead 15-year-old case was reported in 2018. A second elderly case was found in November 2020 in an 18-year-old moose put down due to sickness (Finnish Food Authority). Lastly, 3 cases were identified in female moose (10, 16, 16 years old) in 2019 in Northern Sweden. While the two old females were observed emaciated or showing behavioural changes in the municipality of Arjeplog and Arvidsjaur, the youngest female was shot in the municipality of Arjeplog during the hunting season without signs of illness [41]. A fourth case was reported in September 2020 (14-year-old female) in the county of Västerbotten. This moose was euthanised after being observed walking on three legs only.

Thus, to date in Europe, 34 free ranging CWD cases have been documented in Norway (*N* = 28), Northern Sweden (*N* = 4) and Eastern Finland (*N* = 2). Whereas in reindeer, PrP^CWD^ was detected in the brain and in certain lymphoid organs, an indication of contagiousness, in moose and red deer, PrP^CWD^ was only detected in the CNS. New types of CWD with atypical characteristics were thus considered [33, 35, 39, 41–43].

## No etiological link between North American and Scandinavian CWD strains, potential consequences

As for conventional pathogens, different strains of prions can be identified in the same host species. Prion strains exhibit specific biological traits including time to disease onset, neuropathological patterns of vacuolation and PrP^Sc^ deposition in the brain, and capacity to replicate in the lymphoid tissue. A large body of evidence indicates that prion strain information is encoded within PrP^Sc^ conformation (reviewed in [44]).

A large set of physio-pathological and biochemical criteria can be used to distinguish between prion strains [45]. Among them is the serial transmission to laboratory rodents such as mouse and hamster and the characterization of the disease phenotype. Because of the species barrier that can limit prion transmission from one species to another, prions from naturally infected species may not transmit to laboratory rodents, even at high dose and by intracerebral inoculation. In particular, CWD prions poorly transmit to conventional mice [46]. In this respect, transgenic modelling of animal and human prion diseases by engineering mice to express PrP^C^ from the species of interest has proved incredibly useful for strain typing studies as these models usually lack a transmission barrier against prions from the same species. CWD prions do not escape this rule and propagate in transgenic mouse models expressing cervid PrP. However, so far, the number of CWD strain typing studies has remained relatively rare compared to the number of cases identified and the diversity of species affected, limiting de facto our understanding of the number of strains circulating in a given species and of their capacity to adapt to others.

In a seminal study led by Angers et al. [47], two phenotypically different strains named CWD1 and CWD2 were identified by transmission of a panel of CWD-positive isolates from elk (11 cases), mule deer (16 cases) or WTD (1 case) to cervid PrP mice overexpressing deer PrP^C^ (with Q at codon 226). In deer, these two strains were frequently found to co-propagate. Co-propagation of distinct prion strains is not unusual in TSEs, as shown with human prion strains in CJD affected individuals [48] and with classical scrapie strains in sheep and goats [49].

Non-transgenic laboratory animals can be fairly susceptible to prions from different species, as exemplified by bank voles in which sporadic forms of CJD could be propagated without a transmission barrier [50]. Such studies were instrumental to demonstrate that the force of the species barrier is more a question of conformational compatibility between PrP^C^ and the prion strain type than a species identity [45, 51]. Bank voles (expressing I at codon 109) were also shown to be highly susceptible to CWD prions [52]. Recently, the strain properties of a panel of Canadian CWD isolates (elk, WTD and moose) were compared with those from Norway (reindeer and moose) upon transmission to bank voles. No commonalities between the Canadian and Norwegian isolates were found, in terms of disease tempo on serial passage in bank voles, neuropathology and biochemical properties of PrP^Sc^ that accumulated in the brains of the infected animals. For example, the incubation time to disease at 3^rd^ passage (i.e. when prions are considered adapted to their hosts during cross-species transmission) was very short after inoculation with Canadian CWD prions (35 days) and significantly prolonged and variable after inoculation with Scandinavian isolates (76 (Moose), 105 (Reindeer) and 175 days (Moose)). Further, three different strains were isolated on transmission of CWD-positive Norwegian moose (2) and reindeer (1) [53]. This strain diversity and the absence of etiological link with North American CWD prions was unexpected. In the natural host, polymorphisms at position 95 and 96 from WTD PrP^C^ were shown to impact CWD strain diversification, either by generating new strains or selecting specific conformers [54]. Another polymorphism in this sequence at codon 116 was found to affect prion strain properties, allowing emergence of new strain types [55]. Thus, *PRNP* polymorphisms in *Cervidae* are likely contributors to prion strain diversity and evolution but their impact on the observed differences between North American and Scandinavian isolates remains to be substantiated.

Worryingly, these transmission studies mean that the precise origin of CWD in Europe remains enigmatic and what has been learned from the North American epidemic cannot be readily extrapolated to the European outbreak. Environmental contamination, contagiousness, risks of interspecies transmission and zoonotic potential of European CWD should thus need to be thoroughly assessed.

## Interspecies transmission of CWD

**Figure 1:**
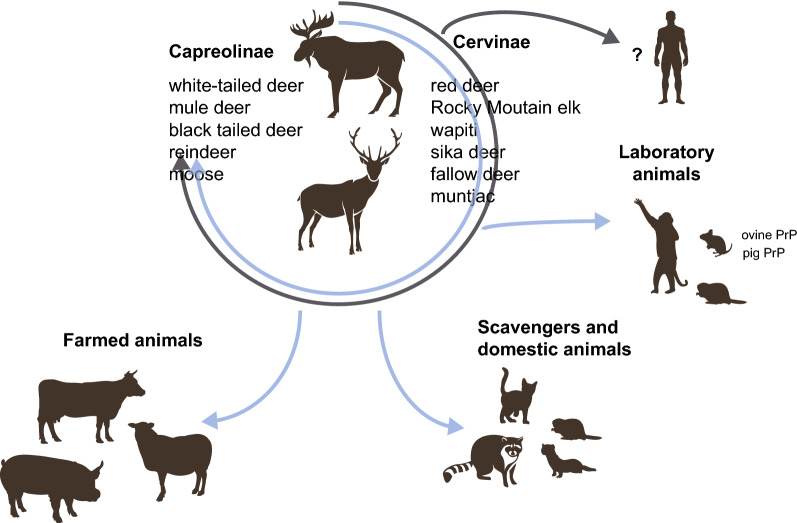
**The host range of CWD prions.** CWD prions circulate in the *Cervidae* reservoir (free-ranging and captive or semi-captive animals). While CWD prion strain diversity extend in the *Cervidae* reservoir is unknown, the strains identified so far in the North-American and European reservoirs are different. North-American CWD prions are able to propagate naturally (grey arrow) or experimentally (blue arrows) in many different species. The impact of intermediate hosts replication on the evolution and zoonotic potential of CWD prions is unknown. Many laboratory species have been experimentally infected with CWD prions, including hamsters, bank voles, transgenic mice expressing ovine or pig PrP and squirrel monkeys. CWD zoonotic threats to humans remain unclear.

The *Cervidae* family includes 40 species of deer that are widely divergent in size, habitat and behaviour. This family is divided into *Capreolinae* and *Cervinae* subfamilies [56]. After natural or experimental infection by oral or intracerebral routes, CWD prions from North America were found to propagate in several members of both subfamilies [22]. Those include WTD (*Odocoileus virginianus*), mule deer (*Odocoileus hemionus*), black tailed deer (*Odocoileus hemionus columbianus*), reindeer (*Rangifer tarandus*) and moose (*Alces alces*) in *Capreolinae* (Figure 1). In *Cervinae,* red deer (*Cervus elaphus elaphus*), Rocky Mountain elk (*Cervus elaphus nelsoni*), wapiti (*Cervus elaphus canadensis*), sika deer (*Cervus nippon*), fallow deer (*Dama dama*) and muntjac (*Muntiacus reevesi*) can be CWD-positive [57, 58].

North American CWD can be experimentally transmitted by intracerebral route to farmed animal species such as pig [59], sheep [60, 61] and cattle [62–66]. By oral route of inoculation, CWD prions are detected in the lymphoid tissue from pig six months after inoculation [59]. Transmission to transgenic mice expressing ovine, and porcine PrP also suggested that CWD prions can propagate in these farm species [67, 68]. In ovinized mice, replication was restricted to the lymphoid tissue, probably because the transmission barrier is lower in this tissue [67]. Furthermore, CWD can be experimentally transmitted to other non-cervid species such as several species of voles [52, 69], white-footed mice [30], Syrian golden hamsters [70], ferrets [71], raccoons [72] and cats [73]. The within- and inter-species transmission potential of CWD is thus relatively large (Figure 1).

So far, no epidemiological evidence supports CWD transmission to human [74]. Non-human primates and transgenic mice expressing human PrP are the most relevant models to address prion zoonotic potential in vivo. Intracerebral and oral inoculation of squirrel monkey with North American CWD induced a typical TSE [11, 75]. Contradictory results have been obtained in macaques, which are considered phylogenetically closer to humans [76, 77]. Humanized transgenic mice did not develop disease after intracerebral inoculation of North American CWD prions [78, 79].

Due to their recent identification, many studies are still ongoing and needed to assess the zoonotic risks associated with the Scandinavian CWD strains and help determining strategies to limit their impact on the wild and farm-populations. Recently, it was shown that humanized mice resisted infection with these agents (primary passage negative) [80]. A larger set of experiments, including

(i) transgenic models in which peripheral replication can be addressed because prion zoonosis can be tissue specific [67],
(ii) a larger panel of CWD isolates from different species because it can impact the transmission properties as exemplified with sheep-passaged BSE [81],

are necessary to conclude on the zoonotic potential of CWD prions.

Besides, some studies focusing on molecular evolution, variability of the prion gene and their effect on the structure of the protein, predicted potential interspecies transmission of CWD. For example, Pronghorn antelopes were predicted to be susceptible to CWD, while bighorn sheep, mountain goats and bison would be more resistant [82]. Experimental demonstration of these predictions remains to be performed. Collectively, these findings highlight that CWD, due to its high proportion to horizontal transfer, to contaminate the ecosystem and to its yet incompletely known zoonotic properties, is a highly problematic ecological, economical, agricultural disease with potential threats to human health.

## Can *PRNP* polymorphism help controlling CWD propagation?

**Figure 2:**
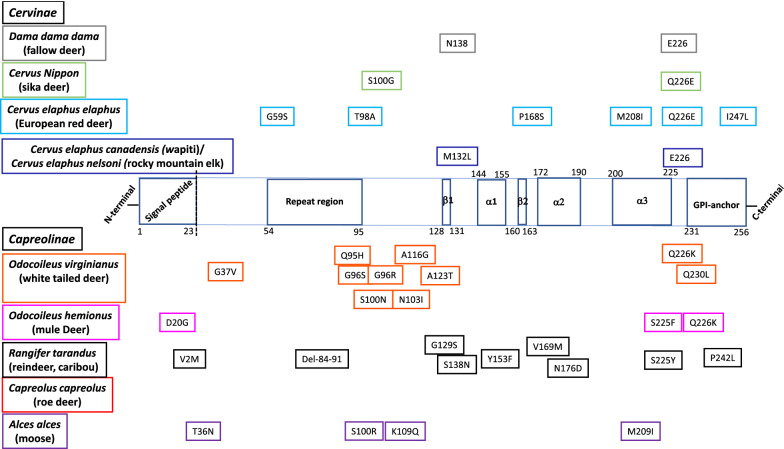
**Amino acid variations within the open reading frame of cervid** ***PRNP*** **placed on the diagram of structural features of elk prion protein.** The numbers from the diagram of structural features have been deduced from the recombinant elk prion protein after nuclear magnetic resonance spectroscopy [125]. The identification in the Protein Data Bank (PDB) entry is PDB ID 1XYW. GPI: Glycosyl-phosphatidyl-inositol anchor, α1, α2, α3: α-strands, β1 and β2 antiparallel β-strands.

Naturally occurring, polymorphisms of the prion protein encoding gene (*PRNP*), an evolutionary well conserved gene in mammalian species, have a direct impact on the susceptibility or resistance to prions. Studies in sheep scrapie have been instrumental in demonstrating the importance of *PRNP* genetics in the etiopathogenesis of the disease. In sheep**,** a range of susceptibility to classical scrapie has been established mainly based on variations at codons A136V, R154H and Q171R, with V_136_R_154_Q_171_ considered the most susceptible haplotype and homozygous A_136_R_154_R_171_ the most resistant. This finding has been used worldwide by many breeding policies to eradicate scrapie [83–85]. In goats, mutations at codons 142 (I/M), 143 (H/R), 146 (N/S), 154 (R/H), 211 (R/Q) and 222 (Q/K) were found to protect against natural scrapie [86–89]. In cattle, indel polymorphisms at the promoter region and intron 1 of *PRNP* were related to an increased BSE incidence [90]. In humans, the M129V *PRNP* polymorphism is strongly associated with variant and sporadic CJD. MV heterozygosity provides relative protection against acquired, sporadic, and some inherited prion diseases. Almost all clinical cases of variant CJD are found in M129 homozygous individuals. Another polymorphism, the G127V provides strong dominant protection against the Kuru disease and diverse prion isolates, as examined by transgenic modelling [91, 92]. In this chapter, we summarize the current knowledge on the potential genetic control of CWD propagation in different cervid populations with an illustration of aa variations within the open reading frame (ORF) of cervid *PRNP* (Figure 2).

### Subfamily *Capreolinae*

#### *Odocoileus virginianus* or white-tailed deer (WTD)

To date, nucleotides variants were observed in WTD at 23 positions; 14 synonymous substitutions at aa positions 20, 51, 81, 95, 108, 124, 126, 139, 146, 147, 156, 166, 185, 202 and 10 non-synonymous substitutions at aa positions G37V, Q95H, G96S or G96R, S100N, N103I, A116G, A123T, Q226K, Q230L encoding 13 different PrP variants. 40 *PRNP* haplotypes were reported (designated A through Z, *PRNP*-Odvi27 to *PRNP*-Odvi38 and AR1, AR2) [93–97].

Very recently, studies with dense sampling allowing formal statistical analysis even on rare *PRNP* alleles were published. One of these studies analysed 9434 farmed WTD from 144 herds in Canada and USA. Among them, 7343 animals were from healthy herds and 2091 from depopulated herds following exposure to CWD with a prevalence rate of 34.1% [93]. Four critical codons were identified at position 95, 96, 116 and 226. At the time of depopulation, 96GG animals were associated with the higher percentage of CWD and the most severe disease stages. 96G/116G, 96GS or 96G/226K and 95H96G had an intermediate position, although for the 96G/226K genotype these differences were not statistically significant. 96SS had the most reduced risk of being CWD-positive and, if positive, were at a significantly earlier stage of disease progression. Still, CWD occurred in 17.5% of 96SS animals. This suggested that 95HH and to a lesser extend 116GG or 226KK homozygotes may have a lowest odds ratios for being found CWD positive with an additive mixed effects model developed to predict outcomes for genotypes with insufficient data [93].

A genetic screening of 2899 free-ranging WTD sampled between 2002 and 2017 from Illinois and Southern Wisconsin with a low CWD prevalence rate of 1% to 2% identified 38 haplotypes [94]. Out of 2754 tested WTD, 407 were CWD-positive and 2347 were CWD-negative. They corresponded to 34 haplotypes and 11 different PrP variants. Seven haplotypes were present at a > 0.01% frequency while the others were rare. These haplotypes correspond to three PrP variants termed: A (Q_95_G_96_S_100_N_103_A_123_Q_226_), C (Q_95_S_96_S_100_N_103_A_123_Q_226_) and F (H_95_G_96_S_100_N_103_A_123_Q_226_), which were detected at a 0.74, 0.17 and 0.05 frequency, respectively. C and F PrP variants were associated with significantly lowered CWD susceptibility compared to A PrP variant, with AA animals showing the highest susceptibility to CWD. F PrP variant had a greater impact than C PrP variant in lowering CWD susceptibility; 3% of CC deer were CWD-positive while no FF or CF deer were identified amongst CWD-positive animals. Variant F effects on prion susceptibility resemble those described for sheep expressing the resistant allele A_136_R_154_R_171_ [98]. A and C PrP variants were similarly identified as the most common *PRNP* haplotypes in 1433 harvested WTD distributed across Arkansas, where CWD prevalence was estimated at 23% in 2015 [95]. They accounted for 82% and 16.71%, respectively. Variant F was absent. Accordingly, A PrP variant was the most frequent within CWD-positive cases. Interestingly, the relative frequency of variant C was over represented in older CWD-positive deer suggesting that this haplotype could slow the disease progression or reduce the likelihood of contracting the disease. Landscape constraints could contribute to a spatial heterogeneity of *PRNP* polymorphisms and impact the prevalence of reduced susceptibility genotypes [95]. Landscape features were also found in 728 free ranging WTD from Mid-Atlantic region, an area with recent history of infection and with low disease incidence [96]. The frequency of susceptible genotypes varied among sub-regions and even among counties within sub-regions separated by large geographical escarpments, large rivers, and/or high-volume traffic roads that influence genetic connectivity. In another study involving 7427 harvested WTD, an inverse relationship between forest habitat and odds of CWD infection was observed in the central Appalachian region of the north-eastern United States where the prevalence rate is 0.93% [99]. It is worth mentioning that the distance of deer dispersal is influenced by the amounts of forest cover, which could affect cross-contamination [100].

Overall, the protective influence of 95H, 96S, 116G and 226K alleles was pointed by different studies. These naturally occurring PrP polymorphisms produced concordant effect with orally inoculated deer [98, 101], transgenic mice expressing deer PrP [35, 47, 102] or during in vitro conversion [103]. Even if deer with protective variants may still be infected with CWD prions, increasing frequency of PrP haplotypes with variants C or F and reducing frequency of variant A may help controlling CWD in WTD [94]. However, the characterization of the infecting strain(s) in these natural conditions was not always assessed. This information is necessary for identifying spill over hosts and estimating the zoonotic potential [53]. Such breeding selection might also contribute to the emergence of new CWD strains [98].

#### *Odocoileus hemionus* or mule deer

To date, nucleotide variants were observed in mule deer at 5 positions; 2 synonymous substitutions at aa positions 131, 247 and 3 non-synonymous substitutions at aa positions D20G, S225F, Q226K [97, 104, 105]. Of note, only two heterozygous animals at codon 226 were identified in Nebraska mule deer (*N* = 122), suggesting a low percentage of animals carrying the 226 K allele.

Under experimental conditions mimicking typical exposure conditions, the 225F allele provided a barrier to infection. After oral inoculation, accumulation and distribution of PrP^CWD^ were similar between 225SF and 225SS deer, but the disease tempo differed. 225SF animals had an extended time to onset of clinical signs compared to their 225SS counterparts [9]. These results are concordant with those obtained when 225SS and 225FF mule deer are cohoused in a contaminated 0.5-Ha paddock [106].

A survey on 1482 free ranging mule deer from Wyoming and Colorado with respect to *PRNP* polymorphisms at codon 225 revealed that animals with 225SS were 30 times more likely to be CWD-positive compared to their 225SF counterparts. No relationship between prevalence rates and genotype frequencies was identified [104]. The protective effect of allele 225F was not observed in Nebraska based on twelve 225SF animals [105]. In another study on 289 unrelated deer from South Saskatchewan (Western Canada), homozygous 20D animals were less likely to be CWD-positive compared to 20GG or 20DG animals [97]. However, there is no definitive correlation between CWD status and PrP polymorphism at codon 20. Indeed, in Nebraska, the 20G allele was significantly associated with reduced odds of being CWD-positive [105] while, in Wyoming and Colorado (*N* = 363) this polymorphism was found to be independent of CWD status [104]. It remains to be evaluated if the same strain was present in all studied animals as different circulating strains may explain these seemingly different results.

Collectively, allele 225F confers a protective effect in mule deer. At the molecular level, this allele has been proposed to induce structural rearrangements in the PrP globular domain, affecting the interaction between α3 helix and the β*2*-α2 loop, and resulting in an increased stability that could interfere with PrP^C^ to PrP^Sc^ conversion rate [47].

### *Rangifer tarandus* (Eurasian wild tundra reindeer, caribou)

To date, nucleotide variants at 9 positions and a 24 bp deletion in the octapeptide repeat region (aa- 84–91) were observed in *Rangifer* species, leading to two synonymous substitutions at aa positions 2 and 146 and to 8 non-synonymous substitutions V2M, G129S, S138N, Y153F, V169M, N176D, S225Y and P242L [34, 107–110].

*PRNP* polymorphisms were studied in the Eurasian wild tundra reindeer (*Rangifer tarandus tarandus*) and in 3 North American caribou subspecies i.e. Alaskan caribou (*Rangifer tarandus granti*), Barren-ground caribou (*Rangifer tarandus groenlandicus*) and woodland caribou (*Rangifer tarandus caribou*), itself subdivided in two major ecotypes, boreal and mountain populations. Due to climate change and industrial development, many Canadian caribou populations are listed as either threatened or endangered. While caribou in Canada are reported free from CWD, some boreal caribou populations have an overlapping habitat with CWD-infected WTD.

Recently, a large scale *PRNP* genotyping was reported from 756 North American caribou sampled in 8 Barren-grounds, 6 mountain woodlands and 7 boreal woodlands caribou herds from two provinces and three western Canadian territories [107]. The analysis focused on the most frequent aa substitutions at positions 129, 138, 146, and/or 169. Rare substitutions at positions 153, 176, 242 and 2 (co-translationally cleaved off) were not considered. Among their pairwise comparisons, only polymorphism at position 138 was significantly different, with the presence of the 138N allele at higher frequency in northern migratory barren ground caribou populations (36.8%) compared to woodland caribous (27.9% and 22.7% in mountain ecotype), when one boreal woodland caribou herd from Chinchaga (63.7%) was excluded. For this latter, landscape features probably explain this high frequency because this herd is located in a habitat surrounded by higher elevation ground that contributes to its geographical isolation. The result from this study is concordant with previous ones conducted at a smaller scale [108, 109]*.*

In central Southern Norway, a genetic screening was performed in 120 Eurasian wild reindeer (*Rangifer tarandus tarandus*). There was 101 healthy animals and 19 CWD-positive cases from Nordfjella zone 1. This resulted in the identification of 5 PrP variants, designated as A (ref sequence: V_2_G_129_S_138_V_169_N_176_S_225_), B (V_2_G_129_S_138_V_169_N_176_Y_225_), C (V_2_del**-**84-91G_129_S_138_V_169_N_176_S_225_), D (V_2_G_129_S_138_V_169_D_176_S_225_) and E (M_2_S_129_S_138_M_169_N_176_S_225_), structured in 14 genotypes [34]. The presence of four instead of five octapeptide repeats is new in Rangifer but was already observed within *Capreolinae* subfamily in *Hydropotes inermis* in Chinese water deer [111]*.* The non-synonymous substitution S138N was not detected, all analysed reindeer being 138SS. Variants A (46.3%) and B (30.4%) were the most common. Variants D and E were not detected among CWD cases. Variant B was more frequent in controls and variants A and C were overrepresented among CWD cases, with A/A and A/C animals presenting a significant CWD risk. These results are concordant with real-time quaking-induced in vitro conversion, where recombinant PrP expressing variants B and E had significantly lower amplification rate than variant A upon conversion with CWD prion seeds [103].

Experimentally, reindeer can contract CWD after oral inoculation with CWD prions from WTD or Elk or after intracerebral inoculation with the aforementioned or mule deer prions. 138SN reindeer have a prolonged incubation period with the absence of typical clinical CWD symptoms at least until 60 months post-inoculation compared to 138SS reindeer, suggesting a partially protective effect of the S138N substitution [112]. 138SN animals had a significant lower lymphoreticular system involvement compared to 138SS and 138NN reindeer [113]*.* In vitro conversion of cervid PrP 138N by CWD seeds was shown to be less efficient than that of cervid *PrP* 138S [114]. However, when housed in contact or adjacent to CWD-infected reindeer, PrP^CWD^ could be found in the lymphoid tissue and brainstem of 138NN animals [113].

Overall, *PRNP* genetic modulations of CWD propagation were identified in reindeer with a protective influence of 138N and of variants B (V_2_G_129_S_138_V_169_N_176_Y_225_), D (V_2_G_129_S_138_V_169_D_176_S_225_) and E (M_2_S_129_S_138_M_169_N_176_S_225_), [34, 103, 112–114]*.* It is possible that the protective effect of these variants will be re-evaluated after characterization of the culled population from Nordfjella (*N* = 2024).

#### *Capreolus capreolus* or roe deer

To date, no polymorphism was found in Roe deer from Great Britain (*N* = 297), Alpine arc of Italy (*N* = 189), Northeast of Spain (*N* = 44) or Sweden (*N* = 11). Only one synonymous substitution at codon 24 was detected in two Swedish animals. This lack of diversity may be due to the relative small number of animals analysed and/or population bottleneck [110, 111, 115, 116].

#### *Alces alces* or moose

To date, nucleotide variants have been identified in moose at 12 positions; 8 synonymous substitutions at aa positions 63, 65, 77, 108, 120, 128, 225 and 243 and 4 non-synonymous substitutions at aa positions T36N, S100R, K109Q and M209I [39, 110, 117–119].

In wild moose, natural CWD infections are rare and one explanation is their tendency to be more solitary than other dense social aggregated *Cervidae*. In Colorado, one affected 209MM moose shot in 2005 and two others shot in 2006 were reported [118]. In Europe, several moose CWD cases were reported, 7 in Norway (including 3 homozygotes moose K_109_ M_209_), 4 in Sweden and 2 in Finland [37, 38, 40, 41].

Following experimental oral inoculation with CWD prions from mule deer, three captive Shira’s moose died without showing any clinical signs indicative of a prion disease. Immunohistochemical evidence for PrP^CWD^ accumulation was observed in a 209MM female that died 465 days post-inoculation and one male that died 113 days post-inoculation. The PrP sequence of the male was not determined because of the lack of suitable tissue for DNA extraction [117]. The third moose that died 567 days after inoculation was negative for PrP^CWD^ and was 209MI heterozygous.

It remains difficult to have a precise estimation of the *PRNP* polymorphisms in moose because of the limited amount of available data. To our knowledge, only data from 163 moose from Alberta, 44 from Alaska, 17 from British Columbia, 15 from Sweden and 7 from Alaska have been published [82, 110, 119].

### Subfamily *Cervinae*

#### *Cervus elaphus canadensis* (wapiti) and *Cervus elaphus nelsoni* (rocky mountain elk)

To date, nucleotides variants were observed in Elk at 3 positions; 2 synonymous substitutions at aa positions 21 and 104 and 1 non-synonymous substitution at aa position 132, M132L, corresponding to the polymorphic position 129 in humans [120–122].

Codon 226 in elk plays a critical role in CWD prion strain selection and PrP^C^ to PrP^Sc^ conversion. Indeed, aa differences at this position controlled (sub)strain selection from different CWD isolates in experimentally inoculated transgenic mouse models [123, 124]. Using a gene-targeted strategy to express physiological levels of PrP^C^ expressing either Q or E at codon 226, which is the only aa difference between mule deer and elk, Bian et al. [123] showed that this polymorphism favoured the selection of either CWD1 (E226) or CWD2 (Q226) conformers in transgenic mice. High resolution nuclear magnetic resonance structure analysis of elk PrP showed that this aa difference could influence the long-range intramolecular interactions and packing of the β2-α2 loop and the C terminus of the α 3 helix of *Cervidae* [47, 125, 126].

After experimental oral inoculation, a 132 *PRNP* genotype-related infection pattern was identified in elk. 132 MM elk developed disease 23 months post-inoculation, ML in 40 months and LL in 59 to 63 months [127, 128]. After intracranial inoculation of groups of Tg12 mice that express M132 elk prion protein, it was suggested that the CWD prion isolated from LL132 elk is a novel CWD strain [129].

A study on 565 elk performed between 2016 and 2018, from a private depopulated land (overall 33% CWD prevalence) from Colorado where CWD was first reported in 2004, showed that 132MM elk were nearly 2 and 3.5 times more likely to be identified as CWD-positive compared to 132ML and 132LL elk, respectively. In addition, 132MM elk were found to be CWD-positive a year sooner, on average, compared to their 132ML counterparts [130].

Interestingly, a recent study described natural adaptation of Elk population to CWD by favouring the 132L allele [131]. A positive correlation between CWD prevalence and the frequency of the 132L allele was found in 1018 elk collected from multiple populations, 3 non-exposed populations (*N* = 533) and 2 populations where CWD was detected 35 years ago (*N* = 485). No additional variants influencing CWD status were observed in a study analysing the promotor region, exons, splice sites, 3’untranslated region, and flanking regions of the *PRNP* gene from 559 captive and free-ranging elk in Colorado, Montana, Minnesota, Nebraska, Oklahoma and South Dakota [122].

#### *Cervus elaphus elaphus* or European red deer

To date, nucleotide variants were identified in red deer at 12 positions; 6 synonymous substitutions at aa positions 15, 21, 63, 78, 79, 136, and 6 non-synonymous substitutions at aa positions G59S, T98A, P168S, M208I, Q226E and I247L.

In Europe*, PRNP* genetic variations are available from 1124 European red deer from 7 Great Britain regions (*N* = 627), 3 Northeast regions of Spain (*N* = 209), Italy (*N* = 191), 6 counties in Norway (*N* = 50 + 1CWD +) and from Western and Eastern lineage from the Czech Republic (*N* = 46) [42, 111, 115, 116, 132]. Three non-synonymous substitutions, G59S, M208I and I247L, and 1 synonymous (position 63) were observed only on single individuals. Four PrP variants were present (T_98_P_168_E_226_, T_98_P_168_Q_226_, A_98_P_168_Q_226_, A_98_S_168_Q_226_) with regional variations between Scotland and/or Northern and/or Southern England.

To our knowledge, only three naturally occurring CWD cases have been reported. One positive case, a near term pregnant female, was found among a captive herd of 500 heads in Minnesota [133]. Another case was reported in a farm in Quebec (Canadian Food Inspection Agency, 2018). One 226 EE was shot in Norway in October 2017 [42]. Under experimental conditions, four red deer (two 226QE, one 226QQ, one 226EE) developed clinical signs of CWD 18 to 20 months after oral inoculation with infectious CWD material from elk [134].

#### *Cervus nippon* or sika deer

Two aa variations have been identified in sequenced sika deer PrP, S100G and Q226E [111, 135–137]. If the E226 and Q226 alleles were equally present in the tested population in China and Korea, the G100 allele was only detected in 3% of the animals, in association with the E226 aa and only the Q226 allele was detected in Europe pure sika. Efficient oral transmission of CWD from Elk to Sika deer was reported [32], but the potential impact of the above-mentioned allelic variations was not tested in this species.

#### *Dama dama dama* or fallow deer

To date, the number of *PRNP* sequences of fallow deer across studies is rather low. The 115 genotypes available are from Great Britain (*N* = 66), Northeast of Spain (*N* = 15), Sweden (*N* = 11) and from an experimental study conducted in Colorado (*N* = 23). Except one synonymous change at codon 138 reported in experimental animals, they all had a single *PRNP* genotype [110, 111, 116, 138]. It seems that fallow deer own a species-specific asparagine (N) at codon 138. Additionally, they have, like elk, a glutamate at codon 226 and this substitution is known to influence the overall protein folding and strain propagation [47, 124–126]. To date, only experimental transmission of CWD to this species has been described [58].

## Conclusion

CWD has spread into wild cervid populations and continues to dramatically increase both in prevalence and geographic range. Among TSEs, CWD has the widest potential species range and its management in free-ranging populations is highly problematic. The number of CWD cases is probably underestimated in Europe and in North America for less highly economically valued species. To date, there is no epidemiological evidence that CWD is associated with human TSEs and no experimental support for its transmission based on limited experimental data with humanized mice. However, more experiments are needed to provide firm conclusions. Furthermore, the risk of novel, potentially zoonotic TSEs via secondary transmission of CWD to farm-species will need dedicated studies [35, 74]. Recently, a list of thirteen groups of risk factors has been established based on their biological plausibility to spread CWD [35], including (i) natural or man-mediated animal aggregation; (ii) fallen stock or inappropriate disposal of carcasses and slaughter by-products; (iii) environmental persistence of prions; (iv) natural movement of live wild deer from infected areas or (v) sex-related behaviours. This disease causes considerable ecologic, economic and sociologic impacts. As illustrated here, *PRNP* sequence availability on large sample size is uneven among *Cervidae.* Generally, game species like white tailed deer, mule deer and elk are more studied. Anyhow, *PRNP* polymorphisms should be considered as key factors that influence CWD susceptibility or disease rate of progression. It seems so far that all deer, irrespective of their *PRNP* genotype, are susceptible to CWD, but natural selection of the less susceptible alleles has been identified. The positive impact of these animals if infected is still a matter of debate since CWD does not compromise reproduction, at least in WTD [28, 139]. CWD positive animals with extended time before they succumb to disease likely represent a source of chronic prion shedding within populations and may contribute to environmental contamination. Many genetic approaches where *PRNP* sequences, genetic relationship, population structure and bottleneck history are used to understand this wildlife disease, but they need to be included into more complex processes. Interactions between hosts, strains and their environment have to be considered. Various CWD strains have already been identified but remain incompletely characterized. CWD can be transmitted horizontally and potentially vertically. Thus, landscape epidemiological studies, combining the fields of landscape ecology with landscape genetics, could foster our understanding and identify factors influencing wildlife dispersal and CWD disease distribution [22, 140]. In the literature, different analytical and statistical methods are proposed for CWD modelling [141]. Recently, a model was provided, based on optimal managing of wildlife populations by using culling to increase disease detection and minimizing undesirable population declines [142]. With an alternative approach of proactive hunting subjected to surveillance, the authors reach 99% probability of freedom from CWD infection of Norwegian reindeer within 3 to 5 years. For this surveillance, a clear infection pattern, selective harvesting and a population model are needed. CWD is a new challenge in wildlife epidemiology that requires multidisciplinary approaches between scientists and stakeholders, including health and governmental authorities [37]. The social aspect and the role of indigenous communities with their cultural practices shall not be neglected.

## Abbreviations

CWD: chronic wasting disease
*PRNP*: prion protein gene
TSE: transmissible spongiform encephalopathy
TSEs: transmissible spongiform encephalopathies
CJD: Creutzfeldt-Jakob disease
PrP^C^: protease-sensitive Cellular PRion Protein
PrP^Sc^: abnormal protease-resistant isoform of the prion protein
BSE: bovine spongiform encephalopathy
CNS: central nervous system
PrP^CWD^: cervid resistant prion protein
WTD: white-tailed deer
PrP: normal cellular Prion Protein
ORF: open reading frame
aa: amino acid
PDB: protein data bank
Gt: gene targeted
